# A phase II, open-label, extension study of long-term patisiran treatment in patients with hereditary transthyretin-mediated (hATTR) amyloidosis

**DOI:** 10.1186/s13023-020-01399-4

**Published:** 2020-07-08

**Authors:** Teresa Coelho, David Adams, Isabel Conceição, Márcia Waddington-Cruz, Hartmut H. Schmidt, Juan Buades, Josep Campistol, John L. Berk, Michael Polydefkis, Jing Jing Wang, Jihong Chen, Marianne T. Sweetser, Jared Gollob, Ole B. Suhr

**Affiliations:** 1 Hospital de Santo António, Centro Hospitalar do Porto, 4099-001 Porto, Portugal; 2 National Reference Centre for Familial Amyloidotic Polyneuropathy (NNERF)/APHP/INSERM U 1195/CHU Bicêtre, 78 rue du Général Leclerc, 94270 Le Kremlin-Bicêtre, France; 3 Hospital de Santa Maria-CHULN, and IMM, Faculdade de Medicina, Universidade de Lisboa, Lisbon, Portugal; 4 Hospital Universitário Clementino Fraga Filho, Federal University of Rio de Janeiro, Ilha do Fundao, Rio de Janeiro, CEP21941-913 Brazil; 5 Universitätsklinikum Münster, Waldeyerstr. 1, 48149 Munster, Germany; 6 Fundació Institut d’Investigació Sanitària Illes Balears (IdISBa), Carretera de Valldemossa, 79, Palma de Mallorca 07120, Balearic Islands, Spain; Servicio de Medicina Interna, Hospital Universitario Son Llàtzer, Carretera Manacor KM, 7198 Palma de Mallorca, Balearic Islands Spain; 7 Hospital Clinic, University of Barcelona, C/ Villarroel, 170, 8036 Barcelona, Spain; 8 Boston University, 72 East Concord Street, K-504, Boston, 02118 USA; 9 Johns Hopkins University, 855 North Wolfe Street, Rangos 435, Baltimore, MD 21205 USA; 10 Alnylam Pharmaceuticals, 300 Third Street, Cambridge, MA 02142 USA; 11 Umeå University, Universitetstorget 16, 901 87 Umeå, Sweden

**Keywords:** ATTR amyloidosis, Cardiomyopathy, Patisiran, Polyneuropathy, RNA interference

## Abstract

**Background:**

Patisiran, an RNA interference therapeutic, has demonstrated robust reduction of wild-type and mutant transthyretin protein and was able to improve polyneuropathy and quality of life following 18 months of treatment in patients with hereditary transthyretin-mediated (hATTR) amyloidosis**.** In this 24-month Phase II open-label extension study, we evaluated the effects of patisiran treatment (0.3 mg/kg intravenously every 3 weeks) on safety, serum transthyretin levels, and clinical parameters. Efficacy assessments included modified Neuropathy Impairment Score +7 (mNIS+7) and multiple disease-relevant measures. Cardiac assessments were performed in a pre-specified cardiac subgroup.

**Results:**

Twenty-seven patients entered this study, including 12 (44%) with ambulation difficulties due to their neuropathy and 11 (41%) who met criteria for the cardiac subgroup. During treatment, the majority of adverse events were mild/moderate in severity; there were no drug-related adverse events leading to treatment discontinuation. The most common drug-related adverse events were flushing and infusion-related reactions (22% each). Patisiran resulted in rapid, robust (~ 82%), and sustained reduction of mean transthyretin levels over 24 months. A mean 6.95-point decrease (improvement) in mNIS+7 from baseline was observed at 24 months. Patisiran’s impact on mNIS+7 was irrespective of concomitant tafamidis or diflunisal use, sex, or age. Clinical assessments of motor function, autonomic symptoms, disease stage, and quality of life remained stable over 24 months. No significant changes were observed for echocardiographic measures or cardiac biomarkers in the cardiac subgroup. Exploratory analyses demonstrated improvements in nerve-fiber density with corresponding reductions in amyloid burden observed in skin biopsies over 24 months.

**Conclusions:**

Long-term treatment with patisiran had an acceptable safety profile and was associated with halting/improvement of polyneuropathy progression in patients with hATTR amyloidosis.

**Trial registration:**

The study was registered at ClinicalTrials.gov (identifier: NCT01961921) on October 14, 2013.

## Background

Hereditary transthyretin-mediated (hATTR) amyloidosis is a progressive and life-threatening disease caused by mutations in the gene encoding the transthyretin (TTR) protein [1, 2]. TTR circulates as a tetrameric protein produced predominantly by the liver [3], with its primary role transporting vitamin A complexed with retinol-binding protein (RBP), with a minor role as a thyroxine carrier [4]. Over 120 *TTR* mutations confer amyloidogenicity [5], resulting in the accumulation of misfolded TTR protein as amyloid fibrils in multiple sites including nerves, heart, gastrointestinal tract, eye, and central nervous system [6, 7]. This pathophysiology leads to a heterogeneous clinical presentation that includes sensory and motor, autonomic, and cardiac symptoms, with the majority of patients displaying a mixed phenotype of both polyneuropathy and cardiomyopathy [8–11]. hATTR amyloidosis is associated with significant morbidity and mortality, with a median survival of 4.7 years following diagnosis [12]. The presence of cardiac involvement is associated with a worse prognosis with a reduced survival (3.4 years) reported in these patients [13].

A substantial decline in quality of life (QOL) and physical functioning is observed as hATTR amyloidosis progresses, reflecting disease involvement across multiple systems. Thus, there is a need for treatments that target the pathogenic TTR protein, rather than the symptoms associated with specific tissues. Current treatment strategies for hATTR amyloidosis directed at disease pathophysiology include prevention of TTR protein production or stabilization of the TTR protein complex. Orthotopic liver transplantation (OLT), which replaces the source of mutant TTR, has been mainly used in patients with early-stage polyneuropathy without cardiac involvement [14]; however, this strategy has been utilized much less in recent years after the introduction of medical therapy [14]. OLT also does not prevent deposition of wild-type (wt) TTR protein, which contributes to subsequent disease progression in post-OLT patients [15]. Treatment with TTR tetramer stabilizers (tafamidis: approved in EU and select regions for treatment of the polyneuropathy of hATTR amyloidosis [16]; and diflunisal: used off-label for hATTR amyloidosis [6]) has been shown to slow progression of neurologic impairment [17, 18]. Additionally, a recent Phase III study of tafamidis showing reduced cardiovascular mortality and hospitalizations in patients with ATTR amyloidosis with cardiomyopathy resulted in USA and EU approval for the treatment of the cardiomyopathy of ATTR amyloidosis derived from wt or mutant TTR for early-stage disease [19]. Inotersen, an antisense oligonucleotide which reduces circulating TTR levels, is approved in the USA and EU for the treatment of the polyneuropathy associated with hATTR amyloidosis [11]. While these treatments may slow the natural progression of hATTR amyloidosis, worsening of neurologic and/or cardiac function is observed in the majority of patients.

Patisiran is an RNA interference (RNAi) therapeutic that targets both wt and mutant *TTR* mRNA, thereby reducing expression of all forms of TTR protein. Patisiran is formulated in a lipid nanoparticle facilitating delivery to the liver, which is the primary site of TTR production [3, 20]. Across the clinical development program, patisiran has demonstrated robust, rapid, and sustained reduction of TTR in patients with hATTR amyloidosis [10, 20, 21]. In the recent Phase III APOLLO study, 18 months of patisiran dosing demonstrated the potential to halt or reverse polyneuropathy and improve QOL from baseline in the majority of patients [10], leading to the approval of patisiran in the USA and several other countries for the treatment of hATTR amyloidosis with polyneuropathy [22, 23]. Patisiran also improved exploratory measures of cardiac structure and function compared with placebo in a pre-specified analysis of patients with cardiac involvement in the APOLLO study [24]. Here, we report the results from an open-label extension (OLE) of a Phase II study of patisiran in patients with hATTR amyloidosis with polyneuropathy. Safety, efficacy, and pharmacodynamic (PD) data are described over 24 months, which is, to date, the longest completed clinical trial with patisiran.

## Methods

### Study design and participants

This was a multicenter, international, Phase II OLE study of patisiran conducted across seven countries: USA, Brazil, France, Germany, Portugal, Spain, and Sweden. Eligible patients had biopsy-proven hATTR amyloidosis with evidence of mild-to-moderate neuropathy, and had previously received and tolerated patisiran in the Phase II study (NCT01617967) [21]. Patients had Karnofsky Performance Status ≥60%; adequate liver and renal function; absolute neutrophil count ≥1500 cells/mm^3^; platelet count ≥100,000 cells/mm^3^; and hemoglobin ≥100 g/l. Key exclusion criteria included: prior OLT; unstable angina or uncontrolled cardiac arrhythmia; and New York Heart Association heart failure class > II. Patients who received an investigational drug other than tafamidis or diflunisal within 30 days of the patisiran first dose were also excluded. Further details on inclusion/exclusion criteria are in Additional file 1: Supplementary methods.

This study was conducted according to the guidelines of the International Conference on Harmonisation, the World Health Organization Declaration of Helsinki, and the Health Insurance Portability and Accountability Act of 1996. Written informed consent was obtained from all participants in the study. The study protocol was approved by local Institutional Review Boards and Ethics Committees, and all subsequent protocol amendments underwent the same approval procedure.

### Procedures

Patients received intravenous infusions of patisiran 0.3 mg/kg (over 70 min) every 3 weeks for approximately 2 years. Patients received a premedication (dexamethasone, paracetamol/acetaminophen, H_1_ and H_2_ blockers, or equivalent) to reduce the likelihood of an infusion-related reaction (IRR). An oral daily supplemental dose of vitamin A was also given. Patients were monitored before the first dose (Day − 28 to Day 0), during dosing (Day 0 to Day 735), and at follow-up (Day 756 and 791). Safety was evaluated throughout the study, and included vital signs, physical and ophthalmologic examinations, clinical laboratory tests, electrocardiograms, and adverse event (AE) monitoring. AEs were categorized by the Investigators as either mild, moderate or severe, and coded according to the Medical Dictionary for Regulatory Activities (MedDRA®) version 18.0.

Serum TTR concentration was assessed using both enzyme-linked immunosorbent assays and turbidimetric assays. Serum RBP was quantified using nephelometry and serum vitamin A was quantified using a high-performance liquid chromatography assay. Details of assessment timings are in Additional file 1: Supplementary methods.

Neurologic function was assessed using the modified Neuropathy Impairment Score +7 (mNIS+7), Neuropathy Impairment Score +7 (NIS+7), and Neuropathy Impairment Score (NIS), at baseline and every 6 months thereafter [25]. To standardize the efficacy assessment and minimize variability across the multiple study centers, neurologists were trained to perform the mNIS+7 evaluation at a central center (Dyck Peripheral Nerve Research Laboratory, Mayo Clinic, Rochester, MN, USA). QOL, disability, motor function, nutritional status, autonomic symptoms, and disease stage (outlined in Outcomes below) were assessed at baseline and every 6 months thereafter (details in Additional file 1: Supplementary methods). Measurements were based on two independent readings taken at least 24 h (but no greater than 7 days) apart and performed by the same investigator when possible. Tandem skin punch biopsies of the distal thigh and distal leg were obtained at baseline and every 6 months thereafter (patient status allowing). These biopsies were used to quantify intra-epidermal nerve-fiber density (IENFD; sensory innervation) and sweat gland nerve-fiber density (SGNFD; autonomic innervation) based on blinded central analysis. Congo Red staining was used to serially assess dermal amyloid burden in the biopsies.

In a subgroup of patients with baseline cardiac involvement (cardiac subgroup; defined as left ventricular [LV] wall thickness of ≥13 mm on echocardiogram in the absence of hypertension, controlled hypertension, or aortic valve disease), serial echocardiograms were performed every 6 months and blood samples were taken every 3 months for central assessment of the cardiac biomarkers troponin I and *N*-terminal pro-brain natriuretic peptide.

### Outcomes

The primary objective was to evaluate the safety and tolerability of long-term dosing with patisiran. Secondary objectives were to assess the PD effect of long-term patisiran dosing on serum TTR levels, and to monitor changes from baseline in the following measures [25]: mNIS+7; QOL (EuroQoL 5-dimensions questionnaire); disability (Rasch-built Overall Disability Scale [R-ODS]); motor function (10-meter [m] walk test and dynamometric grip strength); and nutritional status (modified body mass index). Exploratory endpoints included evaluation of changes in: NIS, NIS+7, familial amyloid polyneuropathy (FAP) stage, polyneuropathy disability (PND) score; patient-reported autonomic symptoms (Composite Autonomic Symptom Score [COMPASS]-31 questionnaire); sensory and autonomic innervation of the skin; dermal amyloid burden [25]; and cardiac parameters (measured by echocardiogram and circulating cardiac biomarkers) in patients in the cardiac subgroup.

### Statistical analysis

For each patient population analyzed in this OLE study, categorical variables were calculated as the number and percentages of patients; for continuous variables, mean, median, standard deviation, and range were calculated. Descriptive statistics provided for clinical laboratory tests, vital signs, all clinical activity parameters, and echocardiograms were presented as actual values and changes from baseline relative to each on-study observation.

This trial is registered with ClinicalTrials.gov, number NCT01961921.

## Results

A total of 27 of 29 patients from the Phase II study [21] were enrolled in this OLE study (1 patient withdrew for personal reasons, the other discontinued the Phase II study due to AEs). The time between last patisiran dose in the Phase II study and first dose in this OLE study ranged from 169 to 512 days. Of the 27 patients enrolled, 25 completed the OLE study (completion date August 31, 2016); 1 patient withdrew after approximately 19 months due to gastro-esophageal cancer (unlikely related to patisiran), which proved fatal, and 1 patient died of a myocardial infarction (not related to patisiran) after completing all dosing but before the end of the study. Median treatment duration was 25 months (range, 19–25 months), with a median of 35 doses (range, 27–36 doses) administered per patient.

Baseline clinical characteristics of the OLE population are shown in Table 1. At study entry, 12 patients (44%) had ambulatory difficulties due to neuropathy (PND score > I), and 20 (74%) and 14 (52%) had a medical history of gastrointestinal or renal and urinary disorders, respectively. Twenty patients (74%) were receiving TTR tetramer stabilizers at baseline; 6 of these patients discontinued stabilizer use (diflunisal [*n =* 5]; tafamidis [*n =* 1]) between 1 and 18 months after entering the study. Eleven patients were included in the cardiac subgroup, for whom the mean LV wall thickness was 1.6 cm (range, 1.3–1.9 cm) (Table 1).

**Table 1:** Patient baseline demographics and disease characteristics

Characteristic	Total population (*n =* 27)
Age, years, median (range)	64 (29 to 77)
Sex, n	
Male	18 (67%)
Female	9 (33%)
Genotype	
Val30Met	20 (74%)
Other^a^	7 (26%)
FAP stage	
1	24 (89%)
2	3 (11%)
PND score	
I	15 (56%)
II	9 (33%)
IIIa	2 (7%)
IIIb	1 (4%)
On TTR stabilizer at study entry	
Tafamidis	13 (48%)
Diflunisal	7 (26%)
None	7 (26%)
mNIS+7 (max. impairment: 304)	53.0 (2.0 to 122.5)
NIS (max. impairment: 244)	34.8 (4.0 to 93.4)
mBMI, (kg/m^2^ × g/L)	1030.5 (728.6 to 1379.6)
EQ-5D (max. impairment: 0)	0.8 (0.3 to 1.0)
Cardiac subgroup	11 (41%)
Val30Met/non-Val30Met^b^, n	8/3
NT-proBNP (pg/mL)^c^	809.8 (105.0 to 2070.0)
Troponin I (ng/mL)^d^	0.14 (0.03 to 0.69)
LV wall thickness (cm)	1.6 (1.3 to 1.9)

*Abbreviations*: *EQ-5D* EuroQoL 5-dimensions questionnaire, *FAP* familial amyloid polyneuropathy, *LV* left ventricular, *mBMI* modified body mass index, *mNIS+7* modified Neuropathy Impairment Score + 7, *NIS* Neuropathy Impairment Score, *NT-proBNP N*-terminal pro-brain natriuretic peptide, *PND* polyneuropathy disability, *QOL* quality of life, *TTR* transthyretin. All data are mean (range) or n (%), unless otherwise stated

^a^Non-Val30Met mutations: Ser77Tyr (*n =* 2), Ser77Phe (*n =* 2), Tyr116Ser (*n =* 1), Phe64Leu (*n =* 1), Arg54Thr (*n =* 1)

^b^Non-Val30Met mutations in cardiac subgroup: Arg54Thr (*n =* 1), Ser77Phe (*n =* 1), Ser77Tyr (*n =* 1)

^c^NT-proBNP normal range is: ≤ 97 pg/mL (age 18–45); ≤ 121 pg/mL (age 45–55); ≤ 198 pg/mL (age 55–65); ≤ 285 pg/mL (age 65–75); ≤ 526 pg/mL (age ≥ 75)

^d^Troponin I normal range is: < 0.03 ng/mL

Overall, 26 patients (96%) reported AEs (Table 2), the majority of which were mild or moderate in intensity. AEs potentially related to patisiran occurred in 16 (59%) patients. No patient discontinued the study due to a drug-related AE. The most common drug-related AEs were mild flushing and IRRs (6 patients [22%] each). The incidence and number of IRRs decreased over time; all IRRs were mild in severity. All patients received their complete dose of patisiran, but 2 patients had temporary infusion interruptions due to IRRs. In 1 patient, the infusion rate was slowed with no subsequent IRRs; the other patient had mild local intravenous site irritation.

**Table 2:** Summary of safety data

AE	Total population (*n =* 27) n (%)
Summary of AEs	
Any AE	26 (96)
Any AE related to study drug	16 (59)
Any serious AE	7 (26)
Any study drug-related serious AE	0
Death	2 (7)
Any AE leading to discontinuation	2 (7)
Common AEs (occurring in > 15% of patients)	
Flushing	7 (26)
Diarrhea	6 (22)
Infusion-related reaction	6 (22)
Nasopharyngitis	6 (22)
Urinary tract infection	6 (22)
Vomiting	6 (22)
Wound	6 (22)
Nausea	5 (19)
AEs related to study drug in > 2 patients	
Infusion-related reaction	6 (22)
Flushing	6 (22)
Diarrhea	3 (11)

*Abbreviation*: *AE* adverse event

Serious AEs were reported in 7 patients (26%), none of which were considered related to patisiran (Table 2). Of the serious AEs, only osteonecrosis (*n =* 2) was reported in more than 1 patient (Additional file 2: Table S1). Severe AEs were reported in 5 patients, with none considered related to patisiran; all severe events were also serious AEs. Two patients died during the study, following cessation of patisiran treatment, as detailed above. There were no clinically significant changes related to patisiran in liver function tests, renal function, thyroid function, or hematologic parameters (including platelet count), and no clinical manifestations of vitamin A deficiency. There were no clinically relevant changes in vital signs, electrocardiogram parameters, or ophthalmology tests. The overall safety profile of patisiran was similar in the cardiac subgroup (Additional file 3: Table S2) compared with the overall study population, and similar irrespective of concomitant TTR tetramer stabilizer therapy (tafamidis and/or diflunisal) (data not shown). Furthermore, the overall safety profiles of concomitant treatment with either patisiran and tafamidis (median exposure 736 days [range: 19–747 days]) or patisiran and diflunisal (median exposure 421 days [range: 139–736 days]) in small cohorts of patients were consistent with the safety profiles of the respective therapies as monotherapies [17, 18].

**Fig. 1:**
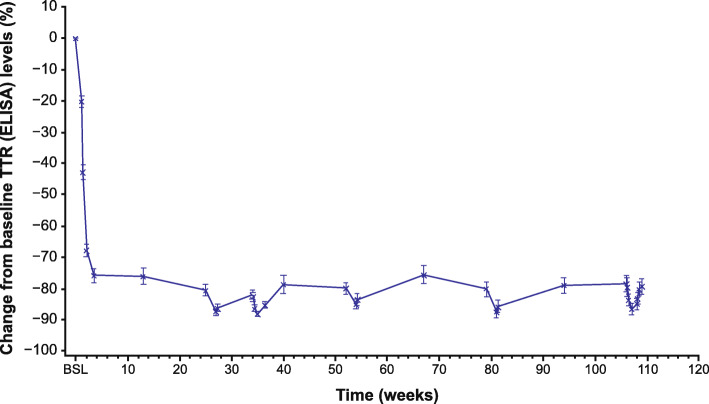
Serum TTR reduction. Percentage change in serum TTR from baseline over time. Pre- and post-dose (0.3 mg/kg Q3W patisiran) values are indicated on the graph with an x. BSL, baseline; ELISA, enzyme-linked immunosorbent assay; Q3W, every 3 weeks; TTR, transthyretin

Serum TTR reduction was rapid and robust following the first dose of patisiran, with mean reduction from baseline nearing 80% by Day 18 (Fig. 1). A sustained mean reduction of 82% was achieved over 24 months, with mean maximal reduction of 93%. The magnitude and dynamics of TTR reduction were similar between patients who received patisiran and TTR stabilizers compared with patisiran alone (Additional file 4: Table S3). Similarly, *TTR* genotype (Val30Met versus non-Val30Met), sex, and age (< 65 versus ≥65 years) did not affect the PD activity of patisiran (Additional file 4: Table S3). Overall, serum vitamin A and RBP levels were reduced by > 65% after 24 months of treatment, as expected based on the known role of TTR in binding and transport of the RBP–vitamin A complex [26].

**Fig. 2:**
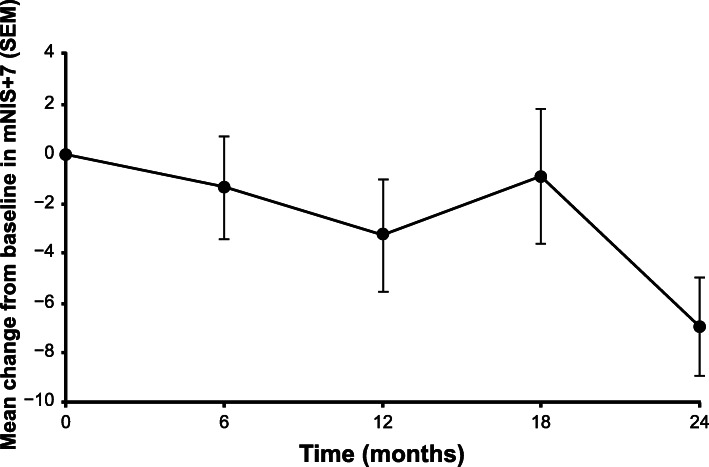
Mean change from baseline (SEM) in mNIS+7 in the all-treated population over 24 months. Error bars represent the SEM. mNIS+7, modified Neuropathy Impairment Score +7; SEM, standard error of the mean

At 24 months, there was a mean mNIS+7 decrease (improvement) from baseline of 6.95 points (Table 3; Fig. 2); 19 patients (70.4%) had an improvement (decrease from baseline at 24 months) in mNIS+7 score. Although patisiran appeared to affect all components of mNIS+7 in a similar manner directionally, sensory measures exhibited the greatest decrease from baseline (improvement) at 24 months by quantitative sensory testing (Table 3). The mean change in mNIS+7 was similar according to TTR tetramer stabilizer use, sex, and in patients < 65 or ≥ 65 years old (Table 3). The mean change (standard error of the mean) in NIS and NIS +7 from baseline at 24 months was + 1.92 (1.84) and + 2.54 (1.84) points, respectively (Additional file 5: Fig. S1a and b).

**Table 3:** Change in mNIS+7 by components and subgroups at 24 months of patisiran treatment

	Change from baseline to Month 24		
	n	Mean (SEM)	Median (range)
mNIS+7 component			
Total	26	−6.95 (2.03)	−7.00 (−34.63 to 15.38)
NIS-Weakness	26	1.23 (1.43)	0.00 (−13.50 to 24.38)
NIS-Reflexes	26	−0.48 (0.53)	0.00 (−6.00 to 7.00)
QST	26	−7.4 (2.04)	−6.0 (−40.0 to 16.0)
NCS ∑5	26	−0.19 (0.18)	−0.25 (−2.00 to 2.50)
Postural BP	26	−0.10 (0.06)	0.00 (−1.00 to 0.50)
mNIS+7 by TTR tetramer stabilizer use			
Patisiran alone	7	−6.75 (5.24)	−8.50 (−28.50 to 15.38)
Patisiran + TTR tetramer stabilizer	19	−7.03 (2.11)	−6.63 (−34.63 to 3.88)

*Abbreviations*: *BP* blood pressure, *mNIS+7* modified Neuropathy Impairment Score +7, *NCS* nerve conduction studies, *NIS* Neuropathy Impairment Score, *QST* quantitative sensory testing, *SEM* standard error of the mean, *TTR* transthyretin

Multiple additional clinical parameters, including EuroQoL 5-dimensions questionnaire, R-ODS, 10-m walk distance, hand grip strength, and COMPASS-31 score, showed stability throughout the 24-month treatment period (Table 4). Within the cardiac subgroup, echocardiographic assessments and serum biomarker levels also remained stable (Table 4). The majority of patients showed stabilization of FAP stage (20 [74%] patients with no change in FAP stage at 24 months) or PND score (23 [85%] patients with no change in PND score at 24 months) (Additional file 6: Table S4).

**Table 4:** Changes in clinical assessments at 24 months with patisiran treatment

Assessment	Change from baseline to Month 24	
	n	Mean (SEM)
EQ-5D (max. impairment: 0)	26	−0.01 (0.02)
R-ODS (no limitations: 48)	25	−1.8 (0.8)
10-m walk (m/s)	21	0.03 (0.04)
Hand grip strength (kg)	26	1.5 (1.2)
mBMI (kg/m^2^ × g/L)	22	−60.8 (34.9)
COMPASS-31 (max. impairment: 100)	26	1.3 (1.8)
Cardiac subgroup (*n =* 11)		
NT-proBNP (pg/mL)	8	−49.6 (170.8)
Troponin I (ng/mL)	8	−0.1 (0.1)
LV mass (g)	10	−16.7 (11.7)
LV wall thickness (cm)	10	−0.08 (0.1)
Ejection fraction (%)	10	−0.6 (1.5)
Peak longitudinal strain (%)	10	0.9 (0.9)
10-m walk (m/s)	7	0.03 (0.05)

*Abbreviations*: *EQ-5D* EuroQoL 5-dimensions questionnaire, *COMPASS* Composite Autonomic Symptom Score, *LV* left ventricular, *m* meter, *mBMI* modified body mass index, *NT-proBNP N*-terminal pro-brain natriuretic peptide, *QOL* quality of life, *R-ODS* Rasch-built Overall Disability Scale

**Fig. 3:**
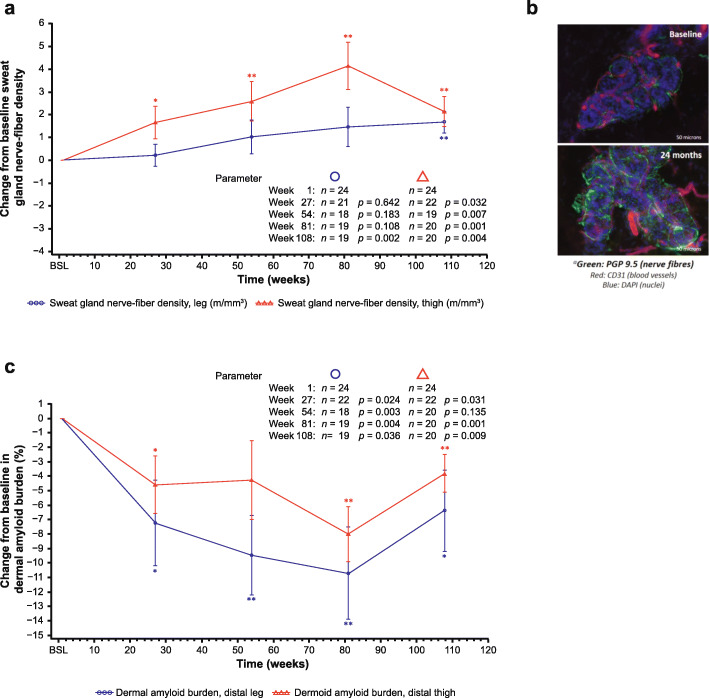
Change in sweat gland nerve-fiber density and dermal amyloid burden of the lower limb. **a** Change in sweat gland nerve-fiber density in distal thigh and distal leg to 24 months. **b** Distal thigh sweat gland innervation at baseline and at Month 24 in an individual patient, with nerve fibers immunostained for PGP 9·5 (green), blood vessels immunostained for CD31 (red), and cell nuclei labeled with DAPI (blue). **c** Change from baseline to 24 months in amyloid burden for the lower limb. The statistical significance (performed as a post hoc analysis) of the change from baseline is shown for each time-point where: *p = 0.01–0.05, **p = 0.001–0.01. BSL, baseline; CD31, cluster of differentiation 31 protein (or platelet endothelial cell adhesion molecule [PECAM1]); DAPI, 4′,6-diamidino-2-phenylindole; PGP, protein gene product

Exploratory analyses evaluating SGNFD of the lower limb demonstrated increased fiber density in the distal thigh at 6, 12, 18, and 24 months, and in the distal leg at 24 months (*p <* 0.05) (Fig. 3a and b). Conversely, a decrease (*p <* 0.05) in dermal amyloid burden was demonstrated in both distal thigh and distal leg over 24 months (Fig. 3c). IENFD remained stable over time (data not shown).

## Discussion

This OLE study evaluated the safety, PD, and clinical effect of patisiran on patients with hATTR amyloidosis with polyneuropathy over 24 months. Patisiran, dosed once every 3 weeks at 0.3 mg/kg, induced rapid and robust TTR reduction in serum. A mean reduction of approximately 82% was sustained across the 24-month treatment period, consistent with the median 81% TTR reduction reported at 18 months in the Phase III APOLLO study. No evidence was found for tachyphylaxis of any PD effect with chronic dosing.

In this study, patisiran was generally well tolerated, with the majority of AEs mild to moderate in intensity, and no patient discontinued treatment due to a patisiran-related AE. Mild IRRs were among the most common drug-related AEs (23%), yet they did not interfere with the completion of dosing, and decreased in incidence over time. No clinically relevant changes in laboratory values related to patisiran, including indicators of liver or kidney function, were observed during the study. In addition, there were no safety signals regarding thrombocytopenia with patisiran [27]. The expected reduction in serum vitamin A levels that accompanied TTR suppression was not associated with any clinical manifestations of vitamin A deficiency (e.g. ocular events), which is possibly due to redundant mechanisms for its transport and tissue uptake [28]. The overall safety profile is consistent with that observed in the APOLLO study [10], with no new safety signals reported with 24 months of patisiran dosing. Furthermore, the safety data in the cardiac subgroup in this study were similar to that observed in the overall population, and with that observed in patients in the cardiac subpopulation in the APOLLO study. Patisiran also had an acceptable safety profile in patients with renal amyloid involvement at baseline, and in patients taking TTR tetramer stabilizers, supporting the potential for patisiran to treat a diverse patient population.

The polyneuropathy seen in symptomatic patients with hATTR amyloidosis is rapidly progressive [29]. While patients with diabetic polyneuropathy exhibit a < 1-point/year increase in NIS-Lower Limbs [30], patients with hATTR amyloidosis and a baseline NIS similar to that in the current study have an estimated 26-point increase in mNIS+7 after 24 months [31]. Indeed, patients in the APOLLO placebo arm demonstrated a 28-point increase in mNIS+7 over 18 months, highlighting the urgency to treat patients with hATTR amyloidosis as early in the disease course as possible [10]. Although the current study did not have a comparator arm, the mean 6*.*95-point improvement in mNIS+7 at 24 months contrasts markedly from the expected worsening without active treatment. Notably, the magnitude of mNIS+7 score improvement in this study is similar to that observed following 18 months of patisiran treatment in APOLLO (6.0-point improvement), supporting the consistent effects of patisiran on polyneuropathy. In this study, these data indicate that patisiran can halt or reverse neuropathy, as 19 of 27 patients showed a decrease from baseline in mNIS+7 at 24 months, demonstrating a reversal in polyneuropathy manifestations in the majority of patients. Similarly, in the APOLLO study the majority of patients demonstrated an improvement in mNIS+7 score compared with baseline [10].

These data are consistent with those reported in APOLLO and compare favorably to those reported for other TTR stabilizing or reduction therapies [11, 17, 18, 32, 33] in patients with hATTR amyloidosis with polyneuropathy. It should be noted that measures of neurologic impairment have varied across studies of different therapeutic agents. The mNIS+7 scale was used in this study and APOLLO [25] to fully assess the progression of this polyneuropathy. The mNIS+7 scale includes measures of autonomic function and nerve conduction that are not captured in the NIS score [34]. Notably, the mNIS+7 utilizes smart somatotopic quantitative sensation testing to provide a better balance between measurement of large and small sensory fibers, and to measure sensation loss across the body [34]. The mNIS+7 scale thus represents a disease-specific measure of polyneuropathy in hATTR amyloidosis, supporting its use as the primary endpoint in recent pivotal studies [10, 11]. In the current study, a consistent, favorable effect on mNIS+7 was observed irrespective of age at onset or mutation status, as was also observed in APOLLO [10].

It should be noted that there was concomitant use of either tafamidis or diflunisal in 20 of 27 patients at baseline. Irrespective of this concomitant TTR tetramer stabilizer use, patisiran was shown to have a similar effect on mNIS+7; notably, the 6 patients who discontinued their stabilizer within 18 months did not exhibit neurologic disease progression.

The decrease in mNIS+7 over 24 months supports the hypothesis that patisiran has the potential to alter the course of hATTR amyloidosis with polyneuropathy. The exploratory findings in autonomic nerve-fiber regeneration and amyloid regression in this study may also highlight a potential effect of patisiran on disease pathophysiology. However, it should be noted that these were exploratory analyses, and that there are currently no natural history data to contextualize the impact of patisiran on nerve-fiber regeneration.

Multiple additional clinical measures stabilized over 24 months, and indeed all measures, whether based on neurologists’ assessments (mNIS+7), patient-based performance (timed 10-m walk), or blinded assessment of pathologic samples (skin biopsy), were similar or improved at 24 months compared with baseline. Additionally, stable COMPASS-31 autonomic symptom scores over the course of the OLE support a stabilizing effect of patisiran on hATTR amyloidosis-related autonomic dysfunction, which is known to impart a substantial disease burden in hATTR amyloidosis. Notably, measures of cardiac structure and function were generally unchanged after 24 months of patisiran dosing in patients with cardiac involvement, contrasting to the worsening of these parameters observed without active treatment. The improvements to neuropathy measures in the cardiac subgroup observed in this study further support the positive effect of patisiran in patients with a mixed phenotype, which was also observed in the APOLLO study [10, 24].

The beneficial effect and tolerability of patisiran treatment, reported here and in the APOLLO study, demonstrate the clinical impact of reducing TTR in patients with hATTR amyloidosis. This extended experience, coupled with secondary analyses of the APOLLO data suggesting benefits in a cardiac subpopulation [24], support examining the effect of patisiran on patients with ATTR amyloidosis with cardiomyopathy (APOLLO-B trial [NCT03997383]) and the development of the investigational RNAi therapeutic vutrisiran. The vutrisiran siRNA utilizes next-generation enhanced stabilization chemistry (ESC), and is conjugated to a trivalent GalNAc ligand to ensure hepatic delivery [35]. The ESC platform is designed for increased potency and high metabolic stability, allowing for a quarterly subcutaneously administered injection that does not require premedication. Expanding the potential range of pharmacotherapies for ATTR amyloidosis will provide greater treatment choice, allowing physicans and patients to select the option most appropriate for this debilitating disease.

## Conclusions

In summary, these results demonstrate that long-term treatment with patisiran had an acceptable benefit:risk profile, and provide further data that it may halt or reverse neuropathy progression as well as have beneficial effects on QOL, physical functioning, autonomic symptoms, and activities of daily living by reducing TTR levels. The safety and efficacy of patisiran is consistent with that reported in APOLLO (NCT01960348), the largest Phase III clinical study to enroll patients with hATTR amyloidosis with polyneuropathy [10]. These data resulted in the approval of patisiran in select countries globally for the treatment of hATTR amyloidosis with polyneuropathy. All 25 eligible patients from this Phase II OLE study have since enrolled in the patisiran Global OLE study (NCT02510261), which will continue to collect long-term safety and efficacy data for an additional 5 years.

## Supplementary information

**Additional file 1.** Supplementary methods

**Additional file 2: Table S1.** Summary of serious adverse events. Seven patients reported a total of 18 serious adverse events.

**Additional file 3: Table S2.** Summary of safety in cardiac subgroup.

**Additional file 4: Table S3.** Summary of mean serum TTR percent reduction by subgroup analysis.

**Additional file 5: Fig. S1.** Mean change from baseline (SEM) in NIS (a) and NIS+7 (b) scores from the all-treated population over 24 months.

**Additional file 6: Table S4.** Change from baseline in PND score and FAP stage at 24 months.

## Data Availability

Authors can confirm that all relevant data are included in the article and its supplementary information files.

## Abbreviations

AE: Adverse event
ATTR: Transthyretin-mediated amyloidosis
BP: Blood pressure
BSL: Baseline
CD31: Cluster of differentiation 31 protein (or platelet endothelial cell adhesion molecule [PECAM1])
COMPASS-31: Composite Autonomic Symptom Score-31
DAPI: 4′;6-diamidino-2-phenylindole
EQ-5D: EuroQoL 5-dimensions questionnaire
FAP: Familial amyloid polyneuropathy
hATTR: Hereditary transthyretin-mediated
IENFD: Intra-epidermal nerve-fiber density
IRR: Infusion-related reaction
LV: Left ventricular
mBMI: Modified body mass index
mNIS: Modified Neuropathy Impairment Score
mNIS+7: Modified Neuropathy Impairment Score +7
NCS: Nerve conduction studies
NIS: Neuropathy Impairment Score
NIS+7: Neuropathy Impairment Score +7
NT-proBNP: *N*-terminal pro-brain natriuretic peptide
OLE: Open-label extension
OLT: Orthotopic liver transplantation
PD: Pharmacodynamic
PGP: Protein gene product
PND: Polyneuropathy disability
Q3W: Every 3 weeks
QOL: Quality of life
QST: Quantitative sensory testing
RBP: Retinol-binding protein
R-ODS: Rasch-built Overall Disability Scale
RNAi: RNA interference
SEM: Standard error of the mean
SGNFD: Sweat gland nerve-fiber density
TTR: Transthyretin
wt: Wild-type
