# Supplementary material for: A phase II, open-label, extension study of long-term patisiran treatment in patients with hereditary transthyretin-mediated (hATTR) amyloidosis
Source: Orphanet J Rare Dis. 2020 Jul 8;15:179. doi: 10.1186/s13023-020-01399-4 (PMC7341568; doi:10.1186/s13023-020-01399-4)
Supplement: Supplementary file 1 — Additional file 1. Supplementary methods [file 13023_2020_1399_MOESM1_ESM.docx]

# Supplementary methods

## Eligibility criteria

For inclusion, patients had Karnofsky Performance Status ≥ 60%; adequate liver function (aspartate transaminase and alanine transaminase ≤ 2.5 × upper limit of normal [ULN], total bilirubin within normal limits, albumin > 30 g/l, and international normalized ratio ≤ 1.2); adequate renal function (serum creatinine ≤ 1.5 ULN); absolute neutrophil count ≥ 1500 cells/mm^3^; platelet count ≥ 100,000 cells/mm^3^; and hemoglobin ≥ 100 g/l. Patients enrolling in the cardiac subgroup had left ventricular wall thickness ≥ 13 mm on transthoracic echocardiogram; were normotensive or had hypertension that was well controlled; and had absence of aortic valve disease.

Key exclusion criteria included: prior orthostatic liver transplant; unstable angina or uncontrolled cardiac arrhythmia; New York Heart Association heart failure class > II; pregnancy; a known or suspected bacterial, viral, parasitic, or fungal infection. Patients who had received an investigational drug other than tafamidis or diflunisal within 30 days of the patisiran first dose were also excluded from the study.

## Pharmacodynamic assessments

For all patients, serum levels of transthyretin (TTR), vitamin A, and retinol-binding protein (RBP) were assessed before dosing at baseline, approximately every 3 months thereafter, and at follow-up. Patients included in the pharmacodynamics subgroup were also assessed for levels of TTR, vitamin A, and RBP after dosing on multiple days following the first dose and following dosing at 8 and 24 months.

## Neurologic and motor function assessments

The modified Neuropathy Impairment Score +7 (mNIS+7) assessment tool is a 304-point composite measure of neurologic impairment which includes: neurologic examination of lower limbs, upper limbs, and cranial nerves (Neuropathy Impairment Score [NIS]-Weakness and NIS-Reflexes); electrophysiologic measures of small and large nerve-fiber function (including nerve conduction studies [NCS] ∑5, ulnar compound muscle action potential [CMAP], ulnar sensory nerve action potential [SNAP], sural SNAP, tibial CMAP, peroneal CMAP, and quantitative sensory testing [QST] by body surface area [including touch pressure and heat pain]); and autonomic function (postural blood pressure). Neurologic impairment was further assessed by the full composite NIS that tests weakness, reflexes, sensation, and cranial nerves. In addition, neurologic impairment was assessed by NIS+7, another composite neurologic impairment score that differs from mNIS+7 in that it uses the full NIS (including NIS-Sensation), does not include QST, has a different NCS Σ5 (sural SNAP, tibial motor distal latency, peroneal CMAP, peroneal motor nerve conduction velocity, and peroneal motor nerve distal latency), includes vibration detection threshold, and uses heart rate response to deep breathing rather than postural blood pressure to monitor autonomic symptoms. Motor function was assessed by timed 10-meter walk and dynamometric hand grip strength, measured at baseline and every 6 months thereafter. Measurements were based on 2 independent readings, and where possible performed by the same investigator. Assessments were performed at least 24 hours (but no greater than 7 days) apart. Patients completed the EuroQoL 5-dimensions questionnaire for quality of life and Rasch-built Overall Disability Score for activity and social function^1^ at baseline and every 6 months thereafter. Nutritional status and autonomic symptoms were also measured every 6 months using modified body mass index (BMI × albumin) and the Composite Autonomic Symptom Score-31 questionnaire,^2^ respectively.

# References

1. van Nes SI, Vanhoutte EK, van Doorn PA, Hermans M, Bakkers M, Kuitwaard K, et al. Rasch-built Overall Disability Scale (R-ODS) for immune-mediated peripheral neuropathies. Neurology. 2011;76(4):337–45.
2. Sletten DM, Suarez GA, Low PA, Mandrekar J, Singer W. COMPASS 31: a refined and abbreviated Composite Autonomic Symptom Score. Mayo Clin Proc. 2012;87(12):1196–201.
