# Supplementary material for: A phase II, open-label, extension study of long-term patisiran treatment in patients with hereditary transthyretin-mediated (hATTR) amyloidosis
Source: Orphanet J Rare Dis. 2020 Jul 8;15:179. doi: 10.1186/s13023-020-01399-4 (PMC7341568; doi:10.1186/s13023-020-01399-4)
Supplement: Supplementary file 2 — Additional file 2: Table S1. Summary of serious adverse events. Seven patients reported a total of 18 serious adverse events. [file 13023_2020_1399_MOESM2_ESM.docx]

Table S1 Summary of serious adverse events. Seven patients reported a total of 18 serious adverse events

| Serious adverse event | Total Population  (n *=* 27) |
| --- | --- |
| At least 1 serious adverse event | 7 (26%) |
| Osteonecrosis^a^ | 2 (7%) |
| Cardiac amyloidosis | 1 (4%) |
| Myocardial infarction | 1 (4%) |
| Abscess limb | 1 (4%) |
| Osteomyelitis | 1 (4%) |
| Urinary tract infection | 1 (4%) |
| Fracture, ankle^a^ | 1 (4%) |
| Fracture, femur^a^ | 1 (4%) |
| Fracture, foot^a^ | 1 (4%) |
| Fracture, tibia^a^ | 1 (4%) |
| Ligament rupture | 1 (4%) |
| Thermal burn | 1 (4%) |
| Dehydration | 1 (4%) |
| Esophageal carcinoma | 1 (4%) |
| Acute prerenal failure | 1 (4%) |
| Arthrodesis^a^ | 1 (4%) |
| Venous thrombosis limb | 1 (4%) |

Data are n (%)

^a^Osteonecrosis was reported with concomitant fractures in both patients, with the osteonecrosis thought to be the results of localized complications that resulted in avascular necrosis and osteochondral injury. One patient, a 40-year-old man, reported fractures of the cuboid and calcaneus of the right foot, a sprain of the tibiotarsal ligament, and rupture of the ligament on Day 48, likely to be due to taking part in a marathon. On Day 233, a computerized tomography scan showed improvement of the ankle fracture with the presence of some bone consolidation, and also showed a fracture of the right internal and external malleolus, an osteochondral defect (osteonecrosis) in the internal dimension of the talus most likely to be due to avascular necrosis, and a fracture at the base of the fourth and fifth metatarsal metatarsals with some bone consolidation. On Day 249, the patient underwent arthroscopic tibio-talo-calcaneal arthrodesis. Subsequently, on Day 428, the patient developed right foot pain due to a technical failure of the arthrodesis and underwent a second arthrodesis of the ankle and sub-astragalus on Day 486. The other patient, a 50-year-old woman, experienced an atraumatic torsion of the right leg on Day 88 and was noted to have a fracture of the medial condyle of the femur with posterior–superior detachment of the fractured bone fragment and fracture of the posterior–medial tibial edge due to an insertion avulsion of the posterior cruciate ligament. During surgical repair, osteonecrosis was noted and thought possibly due to a decreased local vascular supply following the fracture. The investigator thought that systemic corticosteroids might be responsible for the worsening of previous osteopenia, increasing the risk of bone fracture
