# Supplementary material for: A phase II, open-label, extension study of long-term patisiran treatment in patients with hereditary transthyretin-mediated (hATTR) amyloidosis
Source: Orphanet J Rare Dis. 2020 Jul 8;15:179. doi: 10.1186/s13023-020-01399-4 (PMC7341568; doi:10.1186/s13023-020-01399-4)
Supplement: Supplementary file 3 — Additional file 3: Table S2. Summary of safety in cardiac subgroup. [file 13023_2020_1399_MOESM3_ESM.docx]

Table S2 Summary of safety in cardiac subgroup

| Adverse event | Total Cardiac Subgroup Population  (n *=* 11) |
| --- | --- |
| Summary of adverse events Any adverse event Any serious adverse event Any study drug-related serious adverse event | 11 (100%) 3 (27%) 0 |
| Death | 1 (9%) |
| Common adverse events in >15% of patients Flushing Cataract Infusion site extravasation Pyrexia Urinary tract infection Wound Insomnia Myocardial infarction Macular degeneration Diarrhea Fatigue Gait disturbance Infusion site erythema Bronchitis Neuralgia Visual field effect Bronchial disorder Cough | 4 (36%) 3 (27%) 3 (27%) 3 (27%) 3 (27%) 3 (27%) 3 (27%) 2 (18%) 2 (18%) 2 (18%) 2 (18%) 2 (18%) 2 (18%) 2 (18%) 2 (18%) 2 (18%) 2 (18%) 2 (18%) |

Data are n (%)
