# Supplementary material for: A phase II, open-label, extension study of long-term patisiran treatment in patients with hereditary transthyretin-mediated (hATTR) amyloidosis
Source: Orphanet J Rare Dis. 2020 Jul 8;15:179. doi: 10.1186/s13023-020-01399-4 (PMC7341568; doi:10.1186/s13023-020-01399-4)
Supplement: Supplementary file 4 — Additional file 4: Table S3. Summary of mean serum TTR percent reduction by subgroup analysis. [file 13023_2020_1399_MOESM4_ESM.docx]

Table S3 Summary of mean serum TTR percent reduction by subgroup analysis

| Individual mean (SEM) | TTR stabilizer use | | *TTR* genotype | | Sex | | Age group | |
| --- | --- | --- | --- | --- | --- | --- | --- | --- |
|  | TTR stabilizer use  (n *=* 20) | No TTR stabilizer use (n *=* 7) | V30M genotype (n *=* 20) | Non-V30M genotype (n *=* 7) | Male (n *=* 18) | Female (n *=* 9) | < 65 years (n *=* 14) | ≥ 65 years (n *=* 13) |
| TTR percent reduction from baseline over 24 months^a^ | 81.59 (1.36) | 83.41 (3.53) | 81.82 (1.28) | 82.73 (3.84) | 82.05 (1.71) | 82.07 (2.21) | 80.89 (1.67) | 83.31 (2.11) |
| Predose TTR percent reduction from baseline over 24 months^b^ | 78.86 (1.48) | 82.20 (3.75) | 79.39 (1.29) | 80.69 (4.48) | 79.48 (1.89) | 80.22 (2.32) | 78.30 (1.68) | 81.26 (2.42) |
| Maximum TTR percent reduction from baseline over 24 months^c^ | 92.01 (0.77) | 94.05 (1.25) | 92.34 (0.80) | 93.11 (1.23) | 92.85 (0.70) | 91.92 (1.47) | 92.30 (0.70) | 92.80 (1.19) |

*Abbreviations*: *SEM* standard error of the mean, *TTR* transthyretin

^a^For each individual patient the mean TTR % reduction was calculated using all TTR values collected on or after Day 8
^b^For each individual patient the mean predose TTR % reduction was calculated using all TTR values collected predose (within 1 hour prior to planned dosing start) at dosing visits after Day 1
^c^For each individual patient the maximum TTR % reduction was identified
