# Supplementary material for: A phase II, open-label, extension study of long-term patisiran treatment in patients with hereditary transthyretin-mediated (hATTR) amyloidosis
Source: Orphanet J Rare Dis. 2020 Jul 8;15:179. doi: 10.1186/s13023-020-01399-4 (PMC7341568; doi:10.1186/s13023-020-01399-4)

Fig. S1 Mean change from baseline (SEM) in NIS (a) and NIS+7 (b) scores from the all-treated population over 24 months. Error bars represent the SEM. NIS, Neuropathy Impairment Score; NIS+7*,* Neuropathy Impairment Score +7; SEM, standard error of the mean

**a**


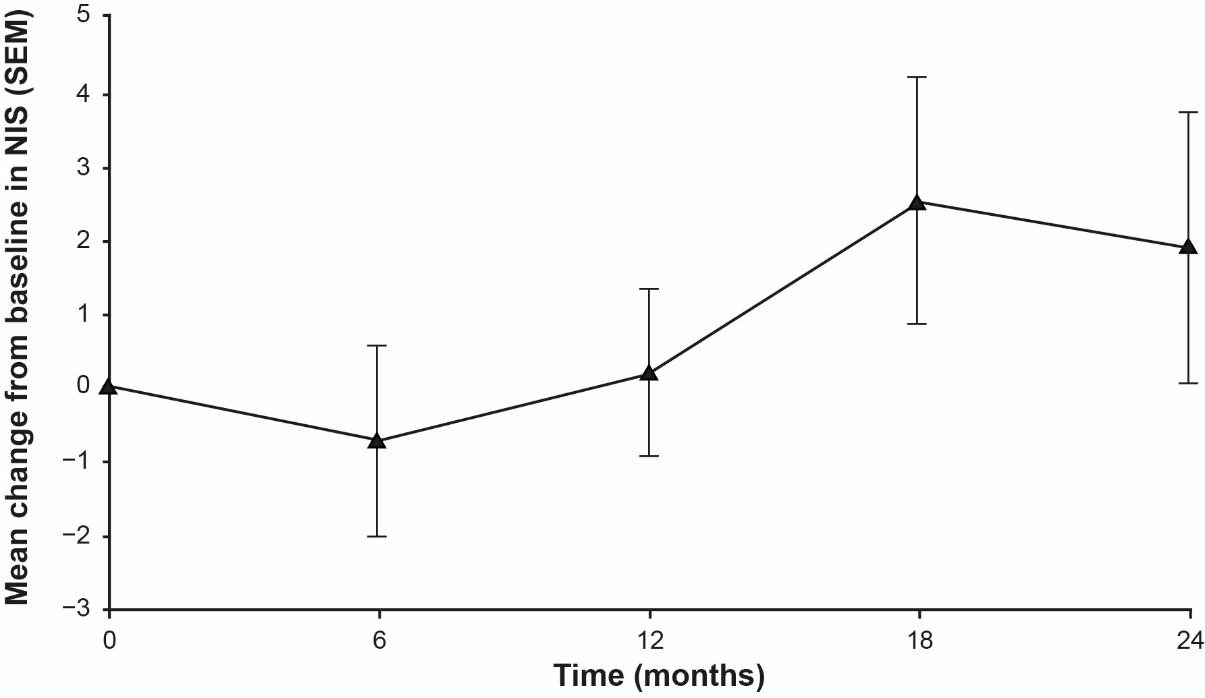


**b**


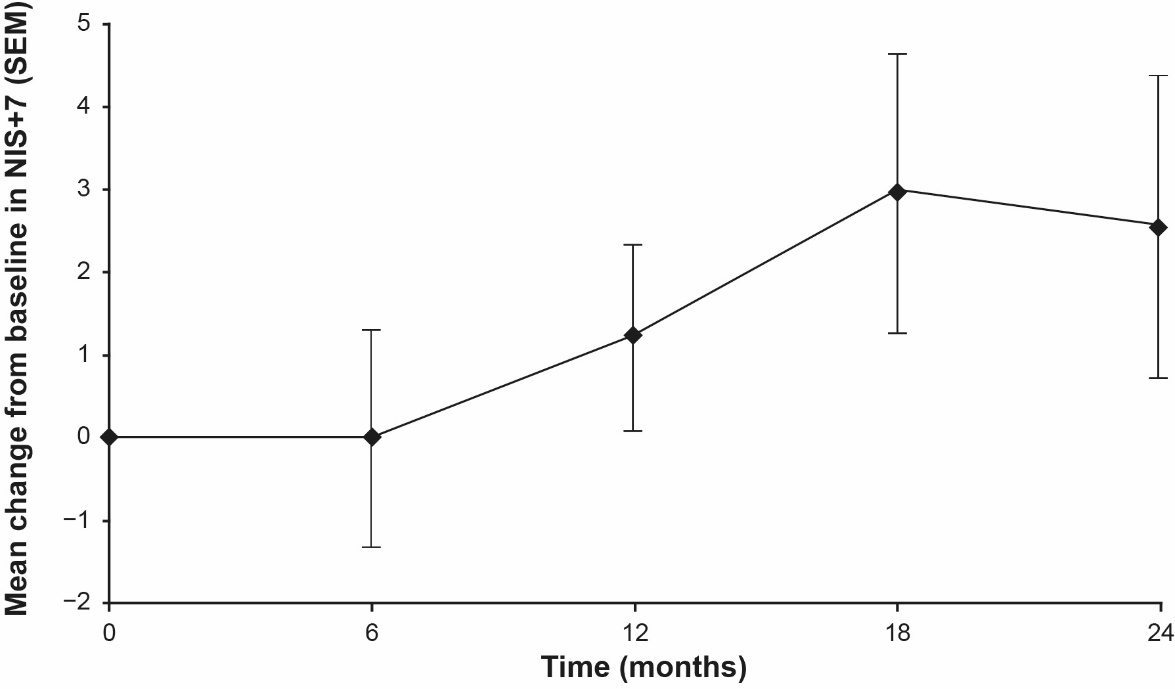

Supplement: Supplementary file 5 — Additional file 5: Fig. S1. Mean change from baseline (SEM) in NIS (a) and NIS+7 (b) scores from the all-treated population over 24 months. [file 13023_2020_1399_MOESM5_ESM.docx]
