# Supplementary material for: A phase II, open-label, extension study of long-term patisiran treatment in patients with hereditary transthyretin-mediated (hATTR) amyloidosis
Source: Orphanet J Rare Dis. 2020 Jul 8;15:179. doi: 10.1186/s13023-020-01399-4 (PMC7341568; doi:10.1186/s13023-020-01399-4)
Supplement: Supplementary file 6 — Additional file 6: Table S4. Change from baseline in PND score and FAP stage at 24 months. [file 13023_2020_1399_MOESM6_ESM.docx]

Table S4 Change from baseline in PND score and FAP stage at 24 months

|  | Value | Stage/score | Overall (n=27) | Any TTR stabilizer use (n=20) | No TTR stabilizer use (n=7) |
| --- | --- | --- | --- | --- | --- |
| PND score | | | | | |
| Baseline | Actual | I II IIIA IIIB IV | 15 (55.6) 9 (33.3) 2 (7.4) 1 (3.7) 0 | 13 (65.0) 5 (25.0) 1 (5.0) 1 (5.0) 0 | 2 (28.6) 4 (57.1) 1 (14.3) 0 0 |
| Month 24 | Actual | I II IIIA IIIB IV | 10 (37.0) 13 (48.1) 2 (7.4) 1 (3.7) 0 | 8 (40.0) 9 (45.0) 1 (5.0) 1 (5.0) 0 | 2 (28.6) 4 (57.1) 1 (14.3) 0 0 |
|  | Comparison | Worsened No change Improved | 5 (18.5) 20 (74.1) 1 (3.7) | 5 (25.0) 13 (65.0) 1 (5.0) | 0 7 (100.0) 0 |
| FAP stage | | | | | |
| Baseline | Actual | 1 2 3 | 24 (88.9) 3 (11.1) 0 | 18 (90.0) 2 (10.0) 0 | 6 (85.7) 1 (14.3) 0 |
| Month 24 | Actual | 1 2 3 | 20 (74.1) 6 (22.2) 0 | 14 (70.0) 5 (25.0) 0 | 6 (85.7) 1 (14.3) 0 |
|  | Comparison | Worsened No change Improved | 3 (11.1) 23 (85.2) 0 | 3 (15.0) 16 (80.0) 0 | 0 7 (100.0) 0 |

Abbreviations: *FAP* familial amyloid polyneuropathy, *PND* polyneuropathy disability, *TTR* transthyretin

Data are n (%)
